# Genome Sequence of a Marine Spirillum, *Oceanospirillum multiglobuliferum* ATCC 33336^T^, Isolated from Japan

**DOI:** 10.1128/genomeA.00396-17

**Published:** 2017-05-25

**Authors:** Joshua G. Carney, Ariel M. Trachtenberg, Bruce A. Rheaume, Joshua D. Linnane, Natalie L. Pitts, Donald L. Mykles, Kyle S. MacLea

**Affiliations:** a Biology Program, University of New Hampshire, Manchester, New Hampshire, USA; b Department of Biology, Colorado State University, Fort Collins, Colorado, USA; c Biotechnology Program, University of New Hampshire, Manchester, New Hampshire, USA; d Department of Life Sciences, University of New Hampshire, Manchester, New Hampshire, USA

## Abstract

*Oceanospirillum multiglobuliferum* ATCC 33336^T^ is a motile gammaproteobacterium with bipolar tufted flagella, noted for its low salt tolerance compared to other marine spirilla. This strain was originally isolated from the putrid infusions of *Crassostrea gigas* near Hiroshima, Japan. This paper presents a draft genome sequence for *O. multiglobuliferum* ATCC 33336^T^.

## GENOME ANNOUNCEMENT

*Oceanospirillum multiglobuliferum* ATCC 33336^T^ (=strain OF1 or IFO 13614^T^) is a Gram-negative, strictly aerobic, motile gammaproteobacterium. *O. multiglobuliferum* (basionym *Spirillum multiglobuliferum* Terasaki 1973) was originally isolated from putrid infusions of the Pacific oyster, *Crassostrea gigas*, on a beach near Hiroshima, Japan, and shows variable morphology, with coccoid bodies predominant after 24 to 48 h of growth (1). Close relatives include *Oceanospirillum linum*, *Oceanospirillum beijerinckii*, *Oceanospirillum maris*, and *Oceanospirillum nioese* (1–4).

*O. multiglobuliferum* is a halophile but has the lowest tolerance for salt in *Oceanospirillum*, growing in 0.5% to 4% (wt/vol) NaCl-containing peptone medium (1). It uses a wide variety of molecules as sole carbon sources, including acetate, propionate, butyrate, succinate, and pyruvate; grows weakly in ethanol, *n*-propanol, and *n*-butanol; and can use ammonium ions as a sole nitrogen source (1).

*O. multiglobuliferum* was obtained from ATCC 33336^T^ in freeze-dried form, rehydrated, and grown in marine peptone broth at 30°C for 72 h at 1 atm. Isolated colonies on marine peptone agar plates were picked and grown in marine peptone broth, and the Genomic-tip 500/G kit (Qiagen, Valencia, CA, USA) was used to prepare genomic DNA (gDNA) from the culture. gDNA was fragmented and tagged using the Nextera DNA library prep kit (Illumina, San Diego, CA, USA) and sequenced on an Illumina HiSeq 2500 instrument, generating 250-bp paired-end reads (Hubbard Center for Genome Studies, Durham, NH, USA). Bioinformatic removal of adapter tag sequences was performed using Trimmomatic (5).

Sequencing resulted in a total of 9,428,123 reads. Reads were assembled using SPAdes version 3.6.2 (6) into 509 total contigs, and the assembly was analyzed using QUAST version 2.3 (7). After annotation with the NCBI Prokaryotic Genome Annotation Pipeline (PGAP) process (8), the assembly had a total length of 3,728,987 bp and an average coverage of 378×. The largest contig was 329,238 bp, with an *N*_50_ value of 149,564 bp. The estimated total genomic G+C content was 45.35%, very close to published values of 46.1% (1) and 45% (3, 9). 

PGAP identified a total of 3,580 genes, 3,413 coding sequences (CDSs), 76 RNA genes, 91 pseudogenes, and 3 clustered regularly interspaced short palindromic repeat (CRISPR) arrays in the *O. multiglobuliferum* genome. Prior to submission of the final version of this paper, another *O. multiglobuliferum* genome was deposited in GenBank by the Joint Genome Institute (JGI) (BioSample SAMN02745127, with project accession no. FUXG00000000 and version number FUXG01000000), which was 3,512,709 bp in length, with an *N*_50_ value of 139,127 bp and 3,141 predicted CDSs. Although lower in predicted CDSs, the richness of the JGI assembly was notably increased: hypothetical proteins were reduced from 1,506 in our assembly to 454 hypotheticals in the JGI genome. Spot-checked genes were found to be identical, consistent with their original provenance from the same ATCC 33336^T^ strain. The completion of 2 quality genomes for *O. multiglobuliferum* will enable effective comparisons with *O. maris* DSM 6286 (BioSample no. SAMN02440652), *O. beijerinckii* DSM 7166 (BioSample no. SAMN02441143), and the recently sequenced *O. linum* (10).

### Accession number(s).

This whole-genome shotgun project has been deposited at DDBJ/ENA/GenBank under the accession no. MTSM00000000. The version described in this paper is version MTSM01000000.
